# X-ray treatment to the face and neck in infancy leading to multiple pathologies in later life: a case report

**DOI:** 10.1186/1757-1626-2-9150

**Published:** 2009-12-06

**Authors:** Paul Dakin

**Affiliations:** 1 Woodlands Medical Practice, 54 Leopold Road, London N2 8BG, UK

## Abstract

**Introduction:**

Therapeutic interventions are made in the best interests of the patient taking into account the risks and benefits believed to be true at the time. However, adverse effects associated with the treatment may only become apparent much later, sometimes long after the anticipated survival of the patient.

**Case presentation:**

A lady in her late sixties presents with multiple pathologies of the head and neck that appear to be a direct consequence of irradiation performed as a young child when she was not expected to live for long. Some deformities resulted early after exposure, but most of the dozen pathologies in the affected area have evolved during the subsequent fifty years.

**Conclusion:**

This case acts as a potent reminder to consider the long term effects of therapeutic ionising radiation, especially if there is a possibility of long term survival post treatment. It also demonstrates the effects of a single physical modality on multiple and wide ranging tissue types, highlighting a continuing need in medical education for the teaching of clinical anatomy and an understanding of histological pathology.

## Introduction

This case demonstrates the serious clinical problems that can develop later in life resulting from deep XR treatment applied to a young child for a skin lesion.

## Case presentation

A-sixty eight-year old Caucasian lady of British Jewish descent presents with the long term sequelae of deep XR treatment administered at the age of six months to the right side of her face. Information regarding the childhood treatment is limited to family testimony as the hospital has closed and the original notes are no longer available. We have no other details regarding dose and frequency of the irradiation. Subsequent health developments and interventions are well documented in the case notes within both the General Practice setting and the hospital departments she has visited since. Relatives, one of whom I interviewed, have told the lady that during the London Blitz, she was treated for an extensive mole on her face and neck that was present at birth, and that being a frail child, she was not expected to live.

**Figure 1:**
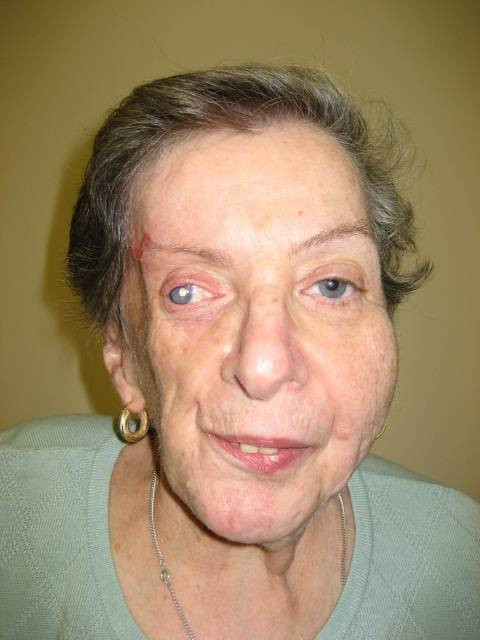
**Full view of the patient's face**.

As a child, right facial asymmetry became progressively more apparent due to arrested development of the skull bones and teeth. (Figure 1) At the age of 47, following complaints of unsteadiness and headaches, a CT scan of the brain demonstrated right-sided cerebellar atrophy with an arachnoid cyst communicating with an enlarged fourth ventricle, and the presence of multiple skull-based meningiomata of the right petrous temporal bone. Within two years, oesophagitis and prepyloric erosions were identified at gastroscopy.

**Figure 2:**
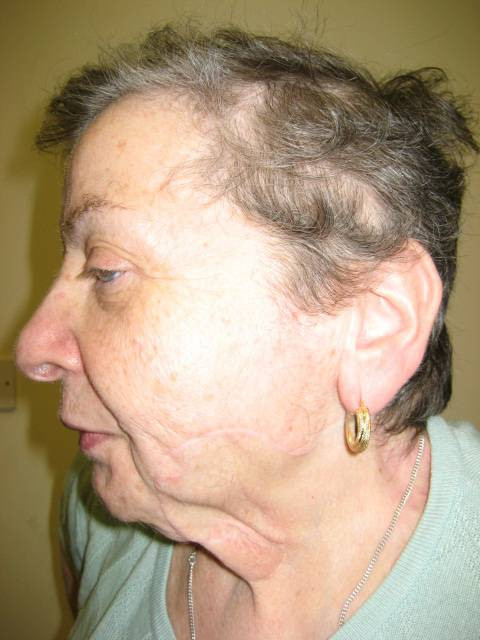
**View of the left side of the patient's face**.

During a four year period from the age of 53, she was diagnosed as having neurofibromata of the left jaw and right cheek; sialadenitis of the left parotid resulting in the gland's excision (as seen in Figure 2) and subsequent Frey's syndrome; bilateral sensorineural hearing loss, worse in the right ear; and an early right cataract.

**Figure 3:**
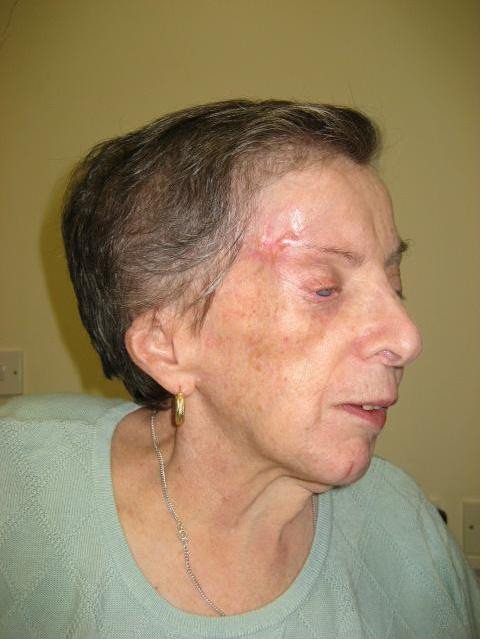
**View of the right side of the patient's face**.

From the age of 60, there were further developments, some more painful and sinister than had occurred previously - right sided trigeminal neuralgia; multiple basal cell carcinomata affecting the right temple (scarring from their excision seen in Figure 3); sarcoma of the left neck recurring two years after excision; a right sided cerebello-pontine angle tumour; and a possible bronchogenic tumour pushing against the oesophagus. The latter two neoplasms have been assessed as inoperable and slow-growing on CT appearance, and the lady has elected not to have further investigation, biopsy or treatment.

As an adult, this lady worked in a leading West End department store until retirement at the normal age. As would be expected, there have been a succession of age related problems, including hypertension, hallux valgus, lumbar spine degeneration, lymphoedema and recurrent varicose veins. At present, she remains a cheerful retired lady, troubled by unsteadiness and gradually worsening headaches, She is kept busy with numerous hospital appointments to attend. She also cares for an older step-sister who suffers from dementia.

## Conclusion

This lady presents with a catalogue of disorders, many of them unusual, affecting a wide set of structures and tissue types, arising predominantly on the right side of the head and neck, corresponding to the area of irradiation in early childhood. Although specific information regarding the original lesion and its treatment is lacking, there is evidence of the use of similar treatment for extensive cutaneous haemangiomata in young children in pre-War Europe, and the subsequent development of clinical pathology [1]. The increased radiation risk is documented [2], with evidence of solid tumour development, particularly of the thyroid and breast [3] and in the central nervous system [4]. It should be noted that there is little literature linking irradiation with the development of arachnoid cysts [5]. Caution has been urged in the use of irradiation in the treatment of benign tumours [6], and the need to reduce those risks when treating childhood malignancies [7].

This case demonstrates how well intended treatment may have severe sequelae that only develop decades later, causing us to reflect on the adequacy of the evidence base for treatment that may be in vogue at any given time. It also reminds us that any health benefits achieved in the short term may be outweighed by subsequent problems that may themselves become life threatening. The unusual nature of the case also emphasises the importance of teaching clinical pathology at a time when many medical schools are dropping the subject from the curriculum, as it demonstrates a single external cause as responsible for the pathological changes in a multiplicity of tissue types.

## Consent

I can confirm that informed written consent was obtained from the patient for publication of the manuscript and figures. A copy of the written consent is available for review by the Editor-in-Chief of this journal.

## Competing interests

The author declares he has no competing interests.
